# Opportunities to develop the professional role of community pharmacists in the care of patients with asthma: a cross-sectional study

**DOI:** 10.1038/npjpcrm.2016.82

**Published:** 2016-11-24

**Authors:** Kim Watkins, Aline Bourdin, Michelle Trevenen, Kevin Murray, Peter A Kendall, Carl R Schneider, Rhonda Clifford

**Affiliations:** 1 School of Medicine and Pharmacology, Centre for Optimisation of Medicines, Pharmacy, The University of Western Australia, Crawley, WA, Australia; 2 Community Pharmacy, School of Pharmaceutical Sciences, University of Geneva, University of Lausanne, Geneva, Switzerland; 3 Centre for Applied Statistics, The University of Western Australia, Crawley, WA, Australia; 4 Faculty of Pharmacy, The University of Sydney, Camperdown, NSW, Australia

## Abstract

There are many indications in Australia and globally that asthma management is suboptimal. Ideally, patients need to proactively self-manage the condition with the support of health professionals. Community pharmacists are a highly accessible resource for patients but currently provide inconsistent services. General practitioners also face many barriers to the provision of chronic disease management for asthma patients. The aim of this research was to characterise patients with asthma who present to community pharmacy. The objective was to identify opportunities to develop the role of pharmacists in the context of the primary healthcare setting and in view of the needs of the patients they routinely encounter. The results of a comprehensive survey of 248 patients recruited from community pharmacies indicated there was discordance between patient perceptions of asthma control and actual asthma control. Almost half the patients surveyed had poorly controlled asthma, whereas almost three quarters perceived their asthma to be well or completely controlled. Fewer than 20% of patients were utilising written asthma action plans, and issues around quality use of medicines were identified. The significance of the incongruent perceptions regarding asthma control is that patients are unlikely to proactively seek intervention and support from healthcare professionals. Community pharmacists provide a significant opportunity to address these issues by direct intervention. There is scope to investigate pharmacists preparing written asthma action plans for patients, using software to monitor medication adherence and prescribe on-going medication. To maximise the potential of pharmacists, barriers to practice need to be identified and addressed.

## Introduction

Despite the important advances made in the past 30 years in the medical management of asthma, the condition remains suboptimally controlled and constitutes a significant health burden.^1,2^ Asthma has impacts at an individual and societal level, and has been a National Heath Priority Area in Australia since 1999.^3^

Currently, asthma is routinely managed in the primary-care setting by general practitioners (GPs). GPs are responsible for writing asthma action plans for patients and prescribing preventer medications (inhaled corticosteroids (ICSs)) to control the condition. However, deficiencies in the quality of care provided by GPs have been observed including, inadequate provision of written asthma action plans, lack of guideline compliant practice and suboptimal patient outcomes.^4–6^ One issue that impedes optimal asthma management by GPs is the lack of routine asthma visits by patients,^4,7,8^ limiting the opportunities for patient education and chronic disease management. GPs observe that patients present only at times of acute exacerbations of asthma.^9^ Meanwhile, community pharmacists are the most highly accessible primary-care health professionals,^10^ yet are an under-utilised resource.^11,12^

Increasingly, there is evidence that indicates the potentially beneficial role that community pharmacists can have in reducing the burden of asthma.^13–16^ However, most of the research to date has been on guided screening and management programmes, undertaken as relatively short-term research interventions.^13,14^ These programmes often require specialised training and resources. There is a paucity of translational research in asthma that develops sustainable roles for community pharmacists that are widely implemented as part of ‘routine practice’ and embraced by the broader healthcare system. On the contrary, there is evidence to indicate that in ‘routine practice’, community pharmacists are not assessing, intervening or referring patients appropriately and are falling well short of their potential.^17,18^ Many barriers are impeding GPs and community pharmacists in the provision of quality, evidence-based care for asthma patients.^6,19–21^

In Australia, the unrealised potential and suboptimal practice by community pharmacists is particularly relevant because of legislation that permits asthma reliever medications to be provided by pharmacists without a prescription. Legally, pharmacists have the responsibility to assess patient therapeutic need and directly supervise the sale of asthma reliever medications under Schedule 3 ‘Pharmacist-Only’ legislation.^22^ The effect of this legislation is that community pharmacists may be the only health professional in a position to regularly assess patients with asthma relying on reliever medications. Even patients using ICSs and/or other prescription medication have prescriptions dispensed by pharmacists on a monthly basis, whereas GPs and medical specialists are able to write a prescription for a 6-month supply and thus may only see the patient twice per year or less, depending upon medical adherence.

In recognition of the unique role that community pharmacists have in asthma management in Australia, the Pharmaceutical Society of Australia endorsed Guidelines for the provision of short-acting β_2_-agonist as Pharmacist-Only medications (SABA guidelines).^23^ However, to develop sustainable roles for pharmacists and identify opportunities for screening, intervention or referral within the scope of routine practice, it is important to understand more about the needs of patients with asthma routinely presenting in community pharmacy. This includes patients obtaining prescriptions as well as those who may be self-managing with reliever medications. There is a need to understand the issues of patients seeking help and advice as well as those who do not acknowledge they have any issues with asthma and simply visit pharmacies as a retail destination. What are the characteristics of the asthma patient that the pharmacist encounters on a daily basis?

The aim of this research was to characterise patients with asthma who present to community pharmacy. The objective was to identify opportunities to develop the role of pharmacists in the context of the primary healthcare setting and in view of the needs of the patients they routinely encounter.

## Results

### Pharmacy and patient numbers

A convenience sample of 50 community pharmacies were invited to participate in the research and 30 agreed to act as patient recruitment sites. Comparisons based on pharmacy location and pharmacy type did not suggest any non-response bias (Table 1). Six Master of Pharmacy students surveyed a total of 249 patients over the 2-month data collection period. The number of questionnaires completed in each pharmacy ranged between 0 and 17 with a mean of 8.3 surveys collected per pharmacy. One survey was excluded due to incomplete data leaving a total of 248 surveys.

### Demographics

Sixty-seven percent of patients surveyed were females, and 72% of patients were born in Australia (Table 2). The survey included an age range of 18.5–93.9 years with a median age of 48.8 years. Data on current employment status were pooled into employed (employed for wages and self-employed) and not employed (out of work and looking, out of work but not looking, homemaker, student, retired, unable to work).

### Patient history and medication use

Of the 248 patients’ surveys, 31 (12.5%) perceived that they had a life-threatening attack in the past 5 years. Thirty-nine patients (15.7%) owned a written asthma action plan to assist in management of their asthma (Table 3).

One hundred and eighty-one patients surveyed (73.0%) were using a ‘preventer’ medication to treat their asthma. Fifty-five patients (22.2%) were managing their asthma with a SABA alone and two patients (0.8%) were using a SABA and long-acting β_2_-agonist (LABA) as their only therapy (Table 4).

Of the 181 patients using ICS, 98 (54.14%) had poorly controlled asthma. There were 148 patients using combination ICS and LABA, and 78 (52.7%) of these had poorly controlled asthma (Table 5). Patients using their asthma reliever inhaler two or more times a week were significantly more likely to be using ICSs than patients not using their reliever that frequently (odds ratio (OR=1.85, 95% confidence interval ((CI)=1.01–3.38, *P*=0.0449).

Table 6 gives a summary of the key results from each of the validated tools incorporated into the Asthma Questionnaire.^24–30^ Using the ACT, patients with a score of 20 or more are considered to have well-controlled asthma, whereas patients with a score of 19 or less are classified as having poor overall asthma control. In this survey, 120 patients (48.4%) had poorly controlled asthma.

### Factors influencing asthma control

Table 7 shows the regression analysis of factors affecting asthma control. Univariate analysis indicated that poor asthma control was significantly related to older age (*P*=0.0068), poor quality of life (*P*<0.0001), poor beliefs about ability to control asthma (*P*<0.0001), use of ICS (*P*=0.0033), smoking (*P*=0.0189) and sinusitis (*P*=0.0065). No relationships were demonstrated between asthma control and knowledge scores or medication adherence scores. Ownership of a written asthma action plan, gender of the patient and concomitant hay fever were also not shown to be predictors of poor asthma control. Multivariate analysis demonstrated that the only significant relationship was between asthma quality of life and asthma control (*P*<0.0001) where patients with higher scores in the AQLQ-S (indicating asthma has a greater negative impact on their quality of life) were significantly more likely to have poorly controlled asthma (for a one-point increase in AQLQ-S score: OR=22.4, 95% CI=9.3–53.9).

### Factors influencing written asthma action plan ownership

A significant positive relationship was found between use of ICS and ownership of a written asthma action plan (ICS use versus no ICS use: OR=2.87, 95% CI=1.07–7.68, *P*=0.036). Variables such as age, sex and asthma knowledge had no relationship with written asthma action plan ownership.

### Factors influencing asthma attacks and emergency medical presentations

Patients with poor asthma control were significantly more likely to have had a ‘life-threatening asthma attack’ in the previous five years compared with those with good asthma control (OR=2.58, 95% CI=1.07–6.22, *P*=0.035). Patients with a greater perceived ability to control asthma were significantly less likely to have had a ‘life-threatening attack’ in the previous 5 years compared with those with poorer perceived ability to control asthma (for a one-point increase in Perceived Control of Asthma Questionnaire score: OR=0.90, 95% CI=0.83–0.97, *P*=0.0039).

Emergency medical presentations included urgent visits to a GP, Emergency Department (ED) or hospital admission, due to asthma getting worse or out of control, or having a life-threatening attack. Patients with poor asthma control were significantly more likely to have had an emergency medical presentation compared with those with good control (OR=2.74, 95%CI=1.59–4.71, *P*=0.0003). Patients with greater perceived ability to control asthma were significantly less likely to have an emergency medical presentation compared with those with poorer perceptions about the ability to control asthma (for a one-point increase in Perceived Control of Asthma Questionnaire score: OR=0.93, 95%CI=0.88–0.98, *P*=0.0055).

## Discussion

### Main findings

This study achieved its aim to provide an understanding of the needs of patients with asthma in the community. It accomplished this through surveying patients in the community pharmacy setting but not targeting any particular subset of patients. In understanding patient need, it was possible to explore how the role of pharmacists can be developed to enhance patient asthma management.

A key finding that warrants further investigation was the discordance between patient perceptions of asthma control and actual asthma control, across the cohort. The significance of these perceptions is that patients are unlikely to proactively seek intervention and support from healthcare professionals in chronic disease management of asthma. They may only access services when experiencing acute exacerbations. Another interesting observation highlighted by the data was that patients had a good understanding of the benefits of written asthma action plans, yet there were low levels of ownership. There was little indication that patients were being proactive in obtaining and using written asthma action plans, despite understanding their importance.

Medication issues were also evident from this survey. There were a significant number of patients with poor asthma control who were using an ICS and, in most instances, also a LABA. Guidelines indicate that most patients can control their asthma symptoms with low-dose ICS.^19,31^ The combination of ICS/LABA is not first-line therapy and is only recommended when medium doses of ICS treatment fail. Given that under-treatment does not seem to be a significant issue related to poor asthma control observed in this cohort, other issues need to be addressed. These could include poor medication adherence, exposure to triggers, symptoms based on co-morbidities, smoking and/or poor inhaler technique.^31^

Although there were a large number of patients being prescribed ICS, there were still over a quarter of patients not using a preventer medication to control their asthma. An explanation for the better control seen in this cohort could be that these patients have less severe disease and hence are able to maintain better control. Nevertheless, there is evidence that patients with persistent asthma but only mild symptoms can benefit from daily ICS treatment.^32^ Meanwhile, the one-third patients not using preventer medication and with poor asthma control are at risk. Excessive use of SABAs has been clearly identified as a risk factor for serious asthma exacerbations and death.^19,33^

A surprising result from this survey was the relatively high proportion of patients who reported having a ‘life-threatening’ asthma attack in the previous 5 years. Life-threatening attacks refer to an ICU admission or the requirement for mechanical ventilation.^19^ Serious sequelae such as these are rare and in Australia in 2008–2009 the overall age-adjusted rate of invasive mechanical ventilation for asthma was just 13.3 per 1,000 hospital separations for asthma.^34^ The statistic measured could be high because it represents the patients’ interpretation of ‘life-threatening attack’. If this were the case, it demonstrates how vulnerable patients feel when they have an acute exacerbation of symptoms. This cohort also indicated high rates of emergency visits to GPs but relatively low rates of ED presentations and hospitalisation. A possible interpretation is that patients have a poor understanding of the symptoms of asthma and when to seek medical help. Another interesting observation, that should be explored further, was the contradictory nature of the relatively high numbers reporting experience of a ‘life-threatening attack,’ compared with the overall low concerns for health, indicated by the quality of life assessments. This observation could support the hypothesis that patients are not concerned and do not ‘pay attention to their asthma’, but panic and are scared when experiencing acute exacerbations.

### Interpretation of findings in relation to previously published work

In this cohort of community pharmacy patients nearly half of the participants (48.4%) were assessed as having poorly controlled asthma, which is consistent with other studies in Australia and overseas.^35,36^ It is lower than the 77% with suboptimal control observed in a cross-sectional study in community pharmacy by Armour and colleagues.^11^ The main difference between the studies is that they were targeting patients for recruitment who were at risk of poor asthma outcomes. The discordance observed in actual asthma control versus perceptions of asthma control was also consistent with much of the literature in Australia^37,38^ and around the world.^35,39^

The accepted issues associated with poorly controlled asthma were also evident from this survey. Patients with poorly controlled asthma were at greater risk of life-threatening attacks, having emergency medical presentations and suffering a reduced quality of life. Contrary to other surveys of asthma patients, no relationships were detected between asthma control and medication adherence and asthma control and rhinitis; however, this may have been due to a lack of power to detect such relationships. The lack of correlation with medication adherence may also have been related to limitations associated with ASK-12 tool, as it is recognised that adherence is difficult to measure.^40^

The ASK-12 is a subjective tool that is reliant on patient memory and willingness to report poor adherence.^27^ Objective measures are the gold standard, and subjective measures are considered less reliable. Problems with reliability were confirmed in validation studies of the ASK-12. The three subscales of the tool were slightly below the accepted cut-offs for reliability (test–retest reliability and internal consistency reliability). It was also noted that reliability might be impacted on in larger samples, because reliability is associated with an upper limit on a scale’s validity.^27^ These factors may all have been relevant to the unexpected results.

Despite the lack of correlation in the overall scores, there were indications from the ASK-12 subscale scores that adherence was not ideal. In one question almost half the patients conceded that they forgot to take their medications sometimes. This is consistent with literature reports that around 50% of patients on long-term therapy fail to take their medication at least some of the time.^19^

It is also well accepted that rhinitis is a co-morbidity that can exacerbate asthma, is frequently under-diagnosed and increasing in prevalence.^41^ The fact that a correlation was not identified in this cohort may also relate to the timing of the survey. Rhinitis is a seasonal condition, and the data collection period for this survey was short and did not coincide with ‘hay fever season’. Conversely, the correlations observed in this cohort between asthma control and sinusitis and asthma control and current smoking were consistent with the literature.^19^

The use of written asthma action plans has been recommended in Australian asthma guidelines for more than 20 years, and initiatives have failed to lift ownership levels that remain unacceptably low. The low levels of ownership seen in this cohort were consistent with Australian data from 2007 to 2008. This data reported 14.4% ownership in persons aged 15 years and over.^34^ In 2013, changes were made to remuneration pathways for GPs, designed to improve chronic disease management and increase levels of written asthma action plan ownership.^42^ However, there is a lack of evidence in this study to demonstrate improvements resulting from these changes.

The medication issues highlighted in this cohort are similar to those in other Australian surveys. In this survey 60% of participants were using ICS/LABA combination therapy, which implies an overuse of expensive and possibly unnecessary medication. High use has been observed in other Australian studies, albeit at variable levels of 50%^36^ and 65%^11^. More disturbing was that this survey and others uncovered a small incidence of LABA use without concomitant ICS treatment. This is contraindicated because of increased risks to morbidity and mortality. The incidence was 0.8% for this cohort with 0.6%^36^ and 4%^11^ in other Australian studies. The high level of ICS in this and other surveys is in contrast to the results from a telephone survey of asthma patients conducted in 2007.^38^ They found the majority (55%) of adult participants were not currently using ICSs, and the figures were even lower in symptomatic patients. The survey also noted that 34% were managing their asthma with SABAs only, which was notably higher than the result of 22% for this survey.^38^

In terms of healthcare utilisation, the results from this survey were inconsistent with those from other Australian surveys. Hospitalisation due to asthma has been reported at levels of around 3.7%,^36,38^ but in this survey the level was 2.8%. Similarly, ED presentations were 10% in a web-based survey but lower in this cohort at 7.7%. In contrast, a high proportion of patients (47%) in this survey reported that they had consulted their GP because their asthma was worse or out of control in the previous 12 months, compared with 23% for the web-based survey. It is likely that these variable results are due to patient interpretation of the questions being asked. In the same web-based survey, 51% reported having a non-urgent visit to a GP for review of asthma, which correlates with this survey result of 47%. It may be that people in the web-based survey understood the term ‘review’ as not being related to chronic disease management, but a ‘review’ of their treatment due to an exacerbation.^36^ Interpretation of survey questions is also likely to have been a factor in patient reports of ‘life-threatening exacerbations’.

### Strengths and limitations of this study

A strength of this study is that it was a survey of community-based patients with asthma, visiting a pharmacy but not necessarily seeking advice or assistance with asthma management. Another strength is the comprehensive nature of the survey, utilising five validated tools to provide a complete picture of patient asthma management. Characterisation of patient attributes and needs permits consideration of the current and potential role community pharmacists can have in intervening to improve supported self-management by patients. It also allows for consideration of the appropriateness of current referral pathways. This research will assist in the development of future research interventions being targeted to patient need and the development of formalised roles for pharmacists in asthma management.

A limitation of the study was the low statistical power for some of the subset analysis. However, given the gaps in the literature, this study was regarded as hypothesis generating research to provide a greater understanding of the needs of patients presenting in community pharmacy. Subsequent research will be fully powered to investigate significant findings. Another limitation was the potential for bias in the recruitment of pharmacies and patients for this study. With a convenience sample, generalisability of the results may be limited. The busy retail environment of community pharmacy meant that staff might not have given all patients with asthma presenting in the pharmacy the chance to participate, resulting in staff-selection bias. There was also the possibility of self-selection bias in the sample. Time-poor patients or those working may not have been well represented. Patients who were concerned about their asthma may have been more willing to participate and over-represented. However, it was notable that the demographics were comparable to larger population studies. Another limitation is one associated with many asthma surveys,^36^ the issue that the diagnosis of asthma was self-reported and not confirmed by medical records. Assessments were only made using self-reported data; inhaler technique of patients was not assessed, which may have an impact on asthma control, even when medication adherence is high.

### Implications for future research, policy and practice

Based on the Pharmacy Guild of Australia figures, the average community pharmacy encounters about 430 patients a month with asthma.^43^ Extrapolating from the results of the questionnaire, community pharmacies on average would encounter 200 patients with poor asthma control per month or 7 per day. The significant cost of these patients to the community in terms of lost productivity and healthcare utilisation is a burden that community pharmacists have the opportunity to reduce. Recent figures for 2015 indicate a total cost for asthma being $28bn a year in Australia.^44^ Hospital admissions, ED presentations and emergency GP presentations could all be reduced by early intervention and referral of patients with poor control by community pharmacists. In the financial year 2008–2009, Australia hospital admission costs alone for asthma totalled AUD $128 million and out of hospital medical services (primarily services provided by registered medical practitioners) totalled AUD $198 million.^45^ Indirect costs associated with lost days off work and study could also be substantially reduced. In 2015, productivity losses due to asthma were estimated to be AUD $1.1 billion.^44^

Currently, health service provision in community pharmacy is inconsistent, and prescription dispensing directs the workflow in most community pharmacies.^46^ Guidelines indicate that pharmacists should refer asthma patients to a GP if they are fulfilling the following: experiencing an acute exacerbation; do not own a written asthma action plan; have not had a medical review in the past 6 months; or have been assessed as having poor asthma control.^23^ From our survey results, these criteria would require pharmacists to refer almost every patient with asthma that they encounter. Clearly, this strategy is not achieving the desired results in terms of asthma control, appropriate use of medicines and written asthma action plan ownership. One way to address the issue is to implement programmes to improve guideline compliant referral by pharmacists; however, this does not tackle the barriers faced at the level of the GP. Another option may be to expand the role of pharmacists. Greater recognition and formalisation of the clinical role of pharmacists may facilitate optimisation of the intervention opportunities for pharmacists as primary healthcare professionals. However, there are many barriers to clinical service provision by pharmacists including low patient receptivity, lack of established inter-professional collaborative pathways, time pressures, organisational issues and remuneration pathways that emphasise the sale of product and efficient dispensing, rather than supporting patient-centred health care.^46–49^ Nevertheless, this option seems reasonable to pursue based on pharmacists proven ability to positively have an impact on health outcomes of asthma patients, their high degree of patient accessibility and the demonstrated patient need.

There are several possibilities for expanded practice by pharmacists. Pharmacists could have a role in the development of written asthma action plans for patients. This addresses the current situation whereby patients are not presenting to doctors for written asthma action plans, despite having a sound understanding of their benefit. For pharmacists to undertake this role, further education and training would be required, but given their expertise in medication, it is within their scope of practice. Trials would need to consider how pharmacists could effectively collaborate with the patient’s doctor to ensure appropriate medical review for patients.

As medication experts, pharmacists are also well placed to monitor the step-up and step-down medication regimen recommended for asthma to optimise therapy. Asthma, as a variable lung condition, requires continual monitoring and reassessment of dosage and this may not be occurring, especially given the low level of written asthma action plan ownership and the high incidence of LABA prescribing observed. Although pharmacists can identify medication issues, to be truly effective at improving outcomes they need to have the capacity to support guideline-based treatment and appropriate patient behaviours. This could possibly occur with an expanded prescribing role.

Under-treatment does not seem to be an issue for many patients in this cohort given the high proportion on combination LABA/ICS. Thus, the poor asthma control observed is likely to be partly attributed to adherence issues including not using the prescribed medications appropriately or poor device technique, despite not being substantiated by this survey. Along with adherence issues, other explanations for poor control include misdiagnosis and difficult-to-treat asthma. It is likely that only a small proportion would fall into these categories in which case the pharmacist could identify and refer these patients. Convenient access and cost of medication can influence ICS use in asthma patients.^31^ Patients may be prescribed ICS initially, but choose to medicate with cheaper and more accessible SABAs, particularly when required to see a GP to obtain on-going ICS prescriptions. They may have a poor understanding of the concept of ‘preventer’ treatments and preferentially choose the treatment that provides obvious and immediate symptomatic relief. ‘Continued Dispensing’ is a novel way that pharmacists could contribute to solving these barriers. The Pharmaceutical Society of Australia released ‘Guidelines for the Continued Dispensing of eligible prescribed medicines by pharmacists V1.0’ in January 2012.^50^ This new legislation allows pharmacists to use their professional judgement to maintain supply of certain medications and ensure continuity of therapy. In this way, pharmacists may intervene when patients seek to self-medicate with SABAs by offering education and a continued supply of ICS. A trial of Continued Dispensing of ICS could be considered given the results of this survey indicating high levels of ICS prescribing but still inadequate asthma control. However, any such trial would need to consider potential detrimental effects to the recommended 6-monthly medical review of patients.

Medication adherence issues are also a key area that pharmacists could have an impact on. Community pharmacists can easily monitor medication adherence via dispensing software. Studies have demonstrated the potential of computer-generated prompts in facilitating pharmacist intervention.^51–53^ The regular contact pharmacists have with patients provides the opportunity for discussion of the complexities that underlie non-adherent behaviour. However, deciphering patient beliefs requires time and advanced communication skills, using patient-centred counselling techniques. Pharmacists may require skills training to undertake more in-depth, motivational style interviewing. Such activities are also labour intensive, and time and resources can also only be allocated given appropriate remuneration.

Although remuneration is an important barrier, it is not the only determinant in pharmacist participation in clinical services, and remuneration alone will not ensure successful uptake.^54^ Inhaler device technique checks are an example that demonstrates this issue. Remuneration has recently become available for pharmacists in Australia for inhaler technique checking,^55^ and this may improve uptake into practice, although other significant barriers to practice change have not been addressed. Workflows based around dispensing and pharmacy layouts possibly contribute to the difficulties in re-organisation of practice to accommodate clinical services and longer patient consultations.^49^ Patient perceptions and receptivity may also be relevant. A lack of expectation of a service may lead to resistance by patients to participate or lack of motivation by pharmacists to provide the service.^46,49^ Multiple expanded services and remuneration options in asthma could provide the necessary incentive to undertake the organisational and capacity changes required. It could also expedite changes in patient perceptions and expectations from a pharmacy consultation. Currently, one of the issues pharmacists have with the non-prescription supply of SABAs are the patient barriers encountered. In a retail environment, patients have a sense of entitlement; they perceive that the medication is safe and do not expect to be asked questions.^9^ These communication issues are further exacerbated and problematic if patients have poor perceptions around asthma control. There are many areas in asthma management and patient care that pharmacists already participate in or could expand their practice to incorporate. These include improving inhaler technique, facilitating smoking cessation and providing holistic patient care by addressing co-morbidities that influence asthma such as rhinitis, sinusitis and depression. The key may be expanding and formalising the pharmacists’ role in asthma to improve the viability of services and ensure consistency of care.

### Conclusions

Pharmacists have the potential to optimise asthma management in the community by direct intervention. Nearly, half of the participants surveyed were assessed as having poorly controlled asthma, yet a proportion of patients displayed a lack of awareness of the issue and were thus unlikely to be seeking support from a health professional. Their poor control had them at risk of life-threatening attacks, requiring emergency medical care and experiencing a reduced quality of life. There is scope to investigate pharmacists preparing written asthma action plans for patients, using software to monitor medication adherence and prescribing on-going medications for asthma, to improve guideline-based management. To maximise the potential of pharmacists, barriers to practice need to be identified and addressed.

## Materials and methods

### Ethics

Ethics approval was obtained from the University of Western Australia’s Human Research and Ethics Committee (HREC RA/4/1/5000) for both the pilot and cross-sectional study. Written informed consent was obtained from all participants in this research.

### Questionnaire development

A questionnaire was designed by the research team to comprehensively characterise patients and their asthma management in the primary healthcare setting. Demographic and patient history questions were based on epidemiological data available in Australia.^34,56^ A review of the literature was undertaken to investigate current, validated tools available. Of primary interest were review articles that provided a comparison and critical appraisal of available tools or recommended a gold standard.^40,57–62^ Also of interest were tools previously used in an Australian setting. Several tools were selected for incorporation into a composite Asthma Questionnaire (Supplementary Appendix 1—Final composite asthma questionnaire—Validated tools included and scoring). Where necessary, licences to use the selected tools were obtained before commencement of study. The finalised Asthma Questionnaire was formatted into a scannable document to improve readability and simplify data entry (Supplementary Appendix 2—Questionnaire). A medication sheet was devised to complement the Asthma Questionnaire and record patients’ current medications. Endorsement of the questionnaire was sought and obtained from the National Secretariat of The Pharmacy Guild of Australia. The questionnaire was given an A1 rating as part of the Survey Approval Program of The Pharmacy Guild of Australia (certificate number 819).^63^

### Pilot study

A pilot study was conducted in a community pharmacy in Perth, Western Australia, between March and May 2012. The aim was to assess the ease of recruiting participants with asthma from community pharmacies and the utility of the new composite Asthma Questionnaire. The pilot identified that patient recruitment was a challenge. Feedback from pharmacy staff and researcher assistants indicated that the length of the questionnaire was a barrier to patient involvement. Researcher assistants administering the questionnaire reported that patients had difficulties in completing the 60-item knowledge section (KASE-AQ—Knowledge, Attitude, and Self-Efficacy Asthma Questionnaire),^64^ and that it substantially added to the time to complete the Asthma Questionnaire.

### Modifications based on pilot study

Changes were made to patient recruitment methodology and to the composite Asthma Questionnaire based on the results of the pilot study. A multimodal method of recruitment was devised to reduce the burden of recruitment on pharmacy staff and to increase the speed of recruitment. Patients were reimbursed for their time. The 60-item KASE-AQ knowledge tool was initially selected for the composite questionnaire based on recommendation in a review article.^62^ It was changed to a more recently developed and shorter tool, the 10-item Consumer Asthma Knowledge Questionnaire (CQ).^29^ The CQ was not originally chosen for the pilot questionnaire due to criticisms raised about an earlier 12-item version.^62^ The authors of the CQ were contacted to obtain more information about the refined 10-item CQ and to respond to the review criticisms that were satisfactorily addressed. Apart from its brevity, another advantage of the CQ over the KASE-AQ was that it was developed by researchers at the University of Sydney and thus highly applicable to the Australian context.^29^

### Cross-sectional survey

The refined composite Asthma Questionnaire was administered as a semi-structured, cross-sectional survey, to patients with asthma, recruited from community pharmacies. The survey was administered to patients between March and May 2013.

### Sample size

The feasibility of patient recruitment was based on information from the Pharmacy Guild Digest that each community pharmacy services on average a population of 4,300 people.^43^ With 10% of the population currently reported to suffer from asthma in Australia^34,45^ that means that each pharmacy provided service to ~430 people with asthma. On average patients visited their community pharmacy about once per month.^43^ Thus, in a 2-week period, an estimate of 215 patients with asthma attended their community pharmacy. Assuming 5% recruitment resulted in a feasibility of 10 patients interviewed per pharmacy over the study period. This number was deemed appropriate and achievable given the resources available for the study. However, it should be noted that no formal priori sample size calculation was carried out.

### Research assistant training

Master of Pharmacy students from the University of Western Australia acted as research assistants to administer questionnaires. They were provided initial training on ethics requirements, background information about asthma, guidelines and validated tools and how to administer the questionnaire. Weekly meetings were held throughout the data collection period to discuss any issues encountered. Research assistants sat with patients as they filled in the questionnaire and could clarify questions but only when patients asked for assistance. For instance, patients were only given definitions of ‘life-threatening asthma’ and ‘written asthma action plan’ if they specifically asked, thus their perception of these concepts was the basis for answers. Research assistants completed the medication sheet with information provided by patients. Following completion of the questionnaire, patients were asked if they had any questions. The research assistants were permitted to offer clinical advice within their capabilities as final year Masters students or could refer the patients back to the pharmacist in store.

### Pharmacy recruitment and training

A convenience sample of community pharmacies, in the north metropolitan area of Perth, Western Australia, was invited to participate in the research as patient recruitment sites. Comparisons based on pharmacy type and pharmacy location were undertaken to check for potential non-response bias. A researcher visited each pharmacy to explain the methodology prior to commencement of the study. A PowerPoint presentation and instruction sheet was used as part of the training. Banners and brochures were also developed to facilitate recruitment and promote the research in store. Research assistants were individually allocated to a pharmacy at set times over a 2-week period for patient interviews and data collection.

### Patient recruitment and inclusion criteria

**Figure 1:**
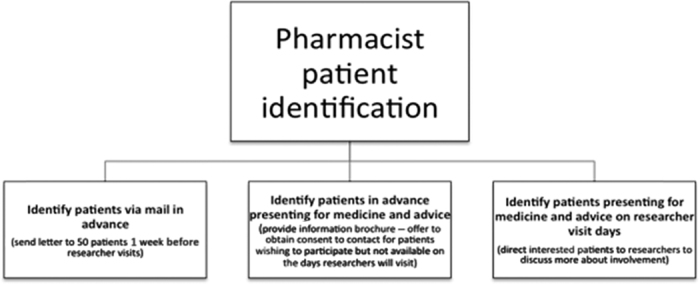
Patient recruitment diagram.

In response to the pilot study, three different recruitment methods were offered to pharmacists to encourage pharmacy participation and to maximise patient interview numbers as per Figure 1. Only one pharmacy decided to send out letters. Most recruitment was by provision of information brochures and incidentally when researchers were in store, where the banners attracted attention. Ethics requirements determined that researchers could not approach patients directly, which added to the challenge of recruitment. Pharmacy staff were instructed to offer interviews to any patient with asthma presenting to the pharmacy. The aim was to schedule interview appointments for at least 10 asthma patients over the 2-week researcher visit. Patient eligibility was based on the following three inclusion criteria: People diagnosed with asthma who were over 18 years of age and able to speak and read English.

### Data analysis

Demographics and medical history were collated and tabulated. Each of the validated tools was scored individually. Summary statistics, including means, s.d., medians, maximums, minimums as well as percentages and counts were calculated for each question and for each tool’s summary score. Binary logistic regression was used to analyse relationships between questionnaire responses and poor asthma control, ICS use, ownership of a written asthma action plan as well as medical presentation and life-threatening attack in the past 5 years (event=‘yes’ for all the analyses). ORs and 95% CIs are provided. General linear regression was used to model (log-transformed) quality of life and (log-transformed) medication adherence and their relationships with patient demographics. Using participants’ postcodes, an Index of Relative Socio-economic Advantage and Disadvantage (IRSAD) value was assigned to each participant. The IRSAD was collected in the 2011 Census of Population and Housing, and is one of the Social Indexes for Areas.^65^ Patient age, sex and IRSAD score were adjusted for in all analyses. For all analyses, variables that were significant at the 5% level were retained in the final model. All data were analysed using the R environment for statistical computing.^66^

## Figures and Tables

**Table 1:** Comparison of pharmacy characteristics between the pharmacies that agreed and refused to participate in the study

	*Pharmacy participated*		*Pharmacy did not participate*	
	N	*%*	N	*%*
*Pharmacy location*				
Street	12	40.00	10	50.00
Medical centre	1	3.33	1	5.00
Shopping centre	17	56.67	9	45.00
				
*Pharmacy type*				
Independent	12	40.00	7	35.00
Chain	18	60.00	13	65.00

**Table 2:** Patient demographics—counts and percentages of categorical patient demographic variables

	N	*%*
*Gender*		
Male	82	33.06
Female	166	66.94
		
*Age (years)*		
18–49	127	51.21
⩾50	121	48.79
		
*Country of birth*		
Australia	179	72.18
Other	69	27.82
		
*Highest level of education*		
Up to year 12	130	52.42
Technical college/Bachelor/Post Graduate	118	47.58
		
*Current employment status*		
Employed	121	48.79
Not employed	127	51.21
		
*Language primarily spoken at home*		
English	239	96.37
Other	9	3.63
		
*Household size*		
Small household (<4 occupants)	186	75.00
Large household (⩾4 occupants)	62	25.00
		
*Income*		
Less than $80,000	155	62.50
$80,000 or more	93	37.50

**Table 3:** Selected responses to patient history questions—counts and percentages of categorical patient history variables

	N	*%*
*Symptoms or treatment for asthma in the past 12 months?*		
Yes	232	93.55
No	16	6.45
		
*Asthma worse or out of control in the past 12 months?*		
Yes	96	38.71
No	152	61.29
		
*Hospital admissions in the last 12 months?*		
Yes	7	2.82
No	241	97.18
		
*Life-threatening asthma attack in past 5 years?*		
Yes	31	12.50
No	210	84.68
Not sure	7	2.82
		
*Days off work, study or usual activities because of asthma?*		
Yes	52	20.97
No	196	79.03
		
*Lifestyle modifications due to asthma?*		
Yes	110	44.35
No	126	50.81
Not sure	12	4.84
		
*Written asthma action plan ownership?*		
Yes	39	15.73
No	209	84.27
		
*Currently smoker?*		
Yes	45	18.15
No	203	81.85
		
*Co-morbidities*—*hay fever?*		
Yes	106	42.74
No	142	57.26
		
*Sinusitis?*		
Yes	41	16.53
No	207	83.47
		
*Depression?*		
Yes	51	20.56
No	197	79.44

**Table 4:** Patient current asthma medications

*Therapy*	N *(%)*
Patients not currently using any medication to control asthma	10 (4.03)
SABA as only therapy	56 (22.58)
SABA and LABA as only therapy (without any ICS)	2 (0.81)
Patients using ICS (with or without other medications)	181 (72.98)
Combination LABA/ICS (with or without other medications)	148 (59.68)
Cromogylycates^a^	3 (1.21)
Montelukast^a^	1 (0.40)
Theophyllines^a^	3 (1.21)

Abbreviations: ICS, inhaled corticosteroid; LABA, long-acting β_2_-agonist; SABA, short-acting β_2_-agonist.

a All patients using these medications were also using an inhaler containing ICS.

**Table 5:** Asthma control and medication use

	*Asthma control*			
	*Poorly controlled*		*Well controlled*	
	N	*%*	N	*%*
*On ICS*				
No	22	32.84	45	67.16
Yes	98	54.14	83	45.86
				
*On ICS/LABA combination*				
No	42	42.00	58	58.00
Yes	78	52.70	70	47.30

Abbreviations: ICS, inhaled corticosteroid; LABA, long-acting β_2_-agonist.

**Table 6:** Key results of individual validated tools from the Asthma Questionnaire

*Asthma control (ACT—Asthma Control Test)*^24,25^	
Mean score of ACT	19.1 (s.d.=4.43, range 5–25)
Median score of ACT	20
Number of patients with poor asthma control	120 (48.4%)
Number of patients who rated their asthma as being well controlled or completely controlled in the previous 4 weeks	175 (70.6%)
Number of patients who thought their asthma was well or completely controlled who were assessed as having good asthma control	119 (68.0% of the 175 patients)
Patients woken at night by asthma in the previous 4 weeks	109 (44.0%)
Patients experiencing shortness of breath at least once in the previous 4 weeks	182 (73.4%)

*Asthma quality of life (AQLQ-S—Asthma Quality of Life Questionnaire*—*Sydney)*^26^	
Mean score of AQLQ-S (adjusted scale 0–10)	1.33 (s.d.=1.54, range 0.00–8.57)
Domain indicating greatest negative impact on quality of life due to asthma	Social disruption domain mean score 2.10 (s.d.=1.99, range 0.00–10.00)
Domain indicating least negative impact on quality of life due to asthma	Concerns for health domain mean score 1.16 (s.d.=1.61, range 0.00–9.64)
Patients who were troubled by shortness of breath in the previous 4 weeks	172 (69.4%)
Shortness of breath that was mildly troubling	102 (41.1%)
Shortness of breath that was severely or very severely troubling	17 (6.9%)
	
*Patient medication adherence (ASK-12*—*Adherence Starts with Knowledge Questionnaire)*^27^	
Mean score of ASK-12	23.4 (s.d.=7.16, range 12–41)
Mean subscale score for inconvenience/forgetfulness	6.92 (s.d.=3.19, range 3–15)
Mean subscale score for treatment beliefs	7.96 (s.d.=7.96, range 4–17)
Mean subscale score for behaviour	8.49 (s.d.=3.50, range 5–22)
Patients who did not disagree with the statement that they forgot to take their medication sometimes	112 (45.2%)
	
*Asthma knowledge (CQ*—*Consumer Asthma Knowledge Questionnaire)*^28,29^	
Mean score of CQ	7.29 (s.d.=1.65, range 2–10)
Mean domain score for management knowledge	4.13 (s.d.=1.17, range 1–6)
Mean domain score for medication knowledge	3.16 (s.d.=0.92, range 0–4)
Patients who knew that written asthma action plans could prevent hospitalisations	216 (87.1%)
Patients with a lack of understanding about medication side effects	139 (56.1%)
	
*Patient beliefs about asthma control (PCAQ*—*The Perceived Control of Asthma Questionnaire)*^30^	
Mean score of PCAQ	43.0 (s.d.=5.57, range 26–55)
Patients who did not disagree with the statement ‘It seems as though fate and factors beyond my control affect my asthma’	102 (41.1%)
Patients who agreed or strongly agreed with the statement ‘If I do all the right things, I can successfully manage my asthma’	221 (89.1%)

**Table 7:** Univariate regression analysis of factors affecting asthma control

*Variables*	*Odds ratio*	*95% Confidence interval*	P*-value*
*Gender*			
Female versus male	1.41	0.83–2.40	0.2072
			
*Age*			
1-s.d. increase (19.63 years)	1.43	1.10–1.84	0.0068
			
*IRSAD*			
1-s.d. increase (39.55 points)	1.16	0.91–1.50	0.2338
			
*Written asthma action plan*			
Yes versus no	1.02	0.51–2.01	0.9641
			
*Overall quality of life score*			
One-point increase	22.35	9.27–53.88	<0.0001
			
*Overall medication adherence scores*			
One-point increase	1.00	0.96–1.03	0.8098
			
*Overall asthma knowledge score*			
One-point increase	0.98	0.84–1.14	0.7955
			
*Patient beliefs score*			
One-point increase	0.89	0.84–0.94	<0.0001
			
*On ICS medication*			
Yes versus no	2.42	1.34–4.35	0.0033
			
*Current smoker*			
Yes versus no	2.23	1.14–4.36	0.0189
			
*Hay fever*			
Yes versus no	1.28	0.77–2.12	0.3410
			
*Sinusitis*			
Yes versus no	2.69	1.32–5.49	0.0065
			
*Combination ICS/LABA*			
Yes versus no	1.54	0.92–2.57	0.0998

Abbreviations: ICS, inhaled corticosteroid; IRSAD, Index of Relative Socio-economic Advantage and Disadvantage; LABA, long-acting β_2_-agonist.
