# Supplementary material for: Opportunities to develop the professional role of community pharmacists in the care of patients with asthma: a cross-sectional study
Source: NPJ Prim Care Respir Med. 2016 Nov 24;26:16082–. doi: 10.1038/npjpcrm.2016.82 (PMC5122313; doi:10.1038/npjpcrm.2016.82)
Supplement: Supplementary Appendix 1 [file npjpcrm201682-s1.doc]

**Appendix 1: Final composite asthma questionnaire – Validated tools included and scoring**

| **Section** | **Chosen validated tool** | **Reason for inclusion** | **Description** | **Scoring** |
| --- | --- | --- | --- | --- |
| **Asthma control** | Asthma Control Test  (ACT)  * Also called the Asthma Score | Five validated tools for the assessment of asthma control were identified and compared. The Asthma Control Test (ACT) was chosen as it offered a quick and easy to administer tool, which had been widely used and tested. It also required no clinical measurements and closely followed the Global Initiative for Asthma (GINA) definition for control. | 5-item questionnaire using a 5-point likert scale.  Items relate to symptoms of asthma; (shortness of breath, night-time symptoms), use of rescue medication, impact on daily functioning and perceived asthma control. | The responses for each question are summated to yield a score ranging from 5-25.  A score of 19 or less indicates partly controlled or poorly controlled asthma. A score of 20 to 25 indicates asthma appears to be controlled. |
| **Quality of life** | Asthma Quality of Life Questionnaire – Sydney  (AQLQ-S) | A review by Apfelbacher identified six quality of life instruments specific for asthma and provided a useful summary to aid selection of a tool for the composite questionnaire. The choice of the Asthma Quality of Life Questionnaire – Sydney (AQLQ-S) was based on its low respondent burden and previous use in an Australian context. | 20-item questionnaire using a 5-point likert scale.  Items cover four domains including: breathlessness, concerns about their asthma, mood and social factors | The total score is the mean of the item scores for all 20 items. Means can also be calculated for each domain.  A higher score indicates a greater impact on quality of life. Scores are multiplied by 2.5 to make the scale between 0 and 10. |
| **Medication adherence** | Adherence Starts with Knowledge Questionnaire  (ASK-12) | The literature acknowledged limitations in the use of self-reported questionnaires for measuring medication adherence and noted that the “gold standard” for adherence measurement is the Medication Events Monitoring System (MEMS®). Nevertheless three validated tools that had previously been used with cohorts of asthma patients were chosen for consideration in the composite questionnaire. The tool chosen was the Adherence Starts with Knowledge (12) Questionnaire (ASK-12) based on its ease of administration, previous use in patients with asthma and use in a community pharmacy setting. | 12-item questionnaire using a 5-point likert scale.  Items in three medication adherence-related subscales including: inconvenience/forgetfulness, treatment beliefs and behaviour. | The total score is the sum of scores for all 12 items and ranges from 12 to 60. Each subscale can also be summated.  A higher score indicates more barriers to medication adherence. |
| **Asthma knowledge** | Consumer Asthma Knowledge Questionnaire (CQ) | No gold standard validated tool, for assessing patient asthma knowledge, was found in the literature. A review article by Pink provided a useful comparison of current asthma knowledge tools but overall was critical of all the available tools and concluded their measures needed to be interpreted with caution. The only tool considered acceptable by Pink was the Knowledge, Attitude, and Self-Efficacy Asthma Questionnaire (KASE-AQ). Furthermore, it had previously been used in an Australian community pharmacy setting. For this reason it was initially chosen for the composite questionnaire. However this choice was reviewed and changed after the pilot study. | 10-item true or false questions.  Items cover two domains including: knowledge of asthma management and knowledge of asthma medications. | The total score is the number of correct responses. Total scores range from 0 to10. Higher scores indicate a better asthma knowledge. |
| **Beliefs, values and perceptions** | The Perceived Control of Asthma Questionnaire (PCAQ) | Many of the knowledge tools identified had domains covering psychosocial factors such as beliefs, perceptions, self-efficacy and attitude including the KASE-AQ. However another specific tool was identified and included in the composite Asthma Questionnaire that looked specifically at perceptions around asthma control. The Perceived Control of Asthma Questionnaire (PCAQ) was chosen for its validated assessment of patient’s self-efficacy, locus of control and learned helplessness. | 11-item questionnaire using a 5-point likert scale  Items cover three domains including: self-efficacy, locus of control and learned helplessness. | The total score is the sum of scores for all 11 items and ranges from 11 to 55.  A higher score indicates greater perceived ability to control asthma. |
