# Supplementary material for: Opportunities to develop the professional role of community pharmacists in the care of patients with asthma: a cross-sectional study
Source: NPJ Prim Care Respir Med. 2016 Nov 24;26:16082–. doi: 10.1038/npjpcrm.2016.82 (PMC5122313; doi:10.1038/npjpcrm.2016.82)
Supplement: Supplementary Appendix 2 [file npjpcrm201682-s2.pdf]

49186

UNIVERSITY OF WESTERN AUSTRALIA

ASTHMA QUESTIONNAIRE

INTRODUCTION

This questionnaire is part of the PARTIES (Patients with Asthma at Risk: Tools Implementation Evaluation Study) pharmacy research supported by the University of Western Australia.

The purpose of this study is to find out more about your asthma and how it impacts on your life. By understanding more about asthma, from people who have the condition, we can:

a. improve health provision
b. help people to better manage their condition.

Your participation in this research will provide valuable information to help further our understanding of the needs of people with asthma.

The information you provide is completely CONFIDENTIAL and will not be linked to you in any way.

By completing and returning this questionnaire, it means that you have given consent for the information you have provided to be used for future research or publishing.

If you require any further information about the study, please do not hesitate to contact:

Ms Kim Watkins
Pharmacy M315
School of Medicine and Pharmacology
Telephone: +61-8-6488-3135
Facsimilie: +61-8-6488-7532
Email: kim.watkins@uwa.edu.au

COMPLETION INSTRUCTIONS

Please take your time, read each question carefully and answer ALL of the questions for each section. If you are not sure of an answer, then ask one of the researchers to help you with it.

Please use a **BLACK** or **DARK BLUE** pen.

Please shade the circles completely (do not tick or cross)	Write clearly within the boxes	Write clearly within each space
●	A B C 1 2 3	PLEASE WRITE IN CAPITAL LETTERS

If you make a mistake, or want to change any of your shaded responses, please place a cross through the incorrect response X and then shade the correct response

For written responses, please cross out your incorrect response and write your new response just above or below the one you have crossed out.

~~I N C O R R E C T~~

CORRECT

SECTION A: PATIENT DEMOGRAPHICS

A1. What is your date of birth?

☐☐ / ☐☐ / ☐☐☐☐

Day Month Year

A2. What is your gender?

- ○ Male
- ○ Female

A3. What is your postcode?

☐☐☐☐

A4. What is your country of birth?

- ○ Australia
- ○ Other (please specify): ______

A5. If born overseas what year did you arrive in Australia?

- ○ Arrival on or before 1985
- ○ Arrival on or after 1986
- ○ Not applicable (i.e. born in Australia)

A6. Are you of Aboriginal or Torres Strait Islands origin?

- ○ Yes
- ○ No
- ○ Not sure

A7. What language do you primarily speak at home?

- ○ English
- ○ Other (please specify): ______

A8. What is your highest level of education?

- ○ Year 10 or below
- ○ Year 11
- ○ Year 12
- ○ TAFE qualification or apprenticeship
- ○ Bachelor's degree
- ○ Post-graduate degree

A9. What is your current employment status?

- ○ Employed for wages
- ○ Self-employed
- ○ Out of work and looking for work
- ○ Out of work but not currently looking for work
- ○ A homemaker
- ○ A student
- ○ Retired
- ○ Unable to work

A10. Including yourself, how many people live within your household?

○ 1 ○ 2 ○ 3 ○ 4 ○ 5 ○ 6 ○ 7 ○ 8 ○ 9 ○ 10+

A11. Please indicate the structure that best defines your household:

- ○ Person living alone
- ○ Couple only
- ○ Couple with children
- ○ Other

A12. What is your total annual household income?

- ○ Less than $20000
- ○ $20000 - $39999
- ○ $40000 - $59999
- ○ $60000 - $79999
- ○ $80000 - $99999
- ○ $100000 - $149999
- ○ $150000 or more

SECTION B: PATIENT HISTORY (ASTHMA, GENERAL MEDICAL AND SOCIAL HISTORY)

B1. At what age were you first diagnosed with asthma? (i.e. told you have asthma by a doctor or a nurse)

☐☐ age in years

B2. Have you had the symptoms of asthma or taken treatment for asthma in the past 12 months?

- ○ Yes
- ○ No

B3. At any time in the past 12 months, was your asthma worse or out of control?

- ○ Yes
- ○ No

B4. In the past 12 months, how many times have you gone to a hospital or emergency department because your asthma was worse or out of control?

☐☐ number of times

B5. In the past 12 months, have you been admitted to hospital due to your asthma?

- ○ Yes
- ○ No
- ○ Not sure

B6. In the past 5 years, have you had a life- threatening asthma attack?

- ○ Yes
- ○ No
- ○ Not sure

B7. In the past 12 months, how many times have you consulted a GP or local doctor because your asthma was worse or out of control?

☐☐ number of times

B8. In the past 12 months, have you had any days off work, study or usual activities because of asthma?

- ○ Yes
- ○ No

B9. Have you made any lifestyle modifications due to your asthma? (e.g. avoidance of certain activities)

- ○ Yes (please specify) ________________
- ○ No
- ○ Not sure

B10. Do you have a written asthma action plan? (i.e. written instructions of what to do if your asthma is worse or out of control)

- ○ Yes
- ○ No

B11. If you have a written asthma action plan do you feel confident to use it in the management of your asthma?

- ○ Yes
- ○ No
- ○ Not sure
- ○ Not applicable (i.e. Do not have an Asthma Action Plan)

B12. Have you previously been given an Asthma Action Plan card?

- ○ Yes
- ○ No
- ○ Not sure

B13. Have you utilized the card as a recording device for medication purchases and/or as a written Asthma Action Plan?
- ○ Yes
- ○ No
- ○ Not sure
- ○ Not applicable (i.e. Have not previously been given an Asthma Action Plan card)

B14. Which of the following best describes your home situation?
- ○ My home is smoke free
- ○ People occasionally smoke in the house
- ○ People frequently smoke in the house

B15. Do you currently smoke?
- ○ Yes
- ○ No

B16. Do you smoke at least once a week?
- ○ Yes
- ○ No
- ○ Not applicable (i.e. Do not smoke at all)

B17. Are you exposed to irritants in the workplace (including cigarette smoke) that exacerbate/trigger your asthma?
- ○ Yes
- ○ No
- ○ Not sure

B18. Do you have any medical history other than asthma? (please choose **ALL** that apply)
- ○ Acne
- ○ Angina
- ○ Anxiety
- ○ Back pain
- ○ Bipolar disorder
- ○ Breast cancer
- ○ Chronic obstructive pulmonary disease (COPD)
- ○ Crohn’s disease
- ○ Depression
- ○ Diabetes
- ○ Eczema
- ○ Emphysema
- ○ Epilepsy
- ○ Glaucoma
- ○ Gout
- ○ Hay fever (allergic rhinitis)
- ○ Heart attack
- ○ Heart failure
- ○ Heartburn
- ○ High blood pressure
- ○ High cholesterol
- ○ Insomnia
- ○ Irritable bowel syndrome
- ○ Kidney disease
- ○ Migraines/headaches
- ○ Obesity
- ○ Osteoarthritis
- ○ Osteoporosis
- ○ Peptic ulcer
- ○ Parkinson’s disease
- ○ Psoriasis
- ○ Reflux disease (GORD)
- ○ Rheumatoid arthritis
- ○ Sinusitis
- ○ Sleep apnoea
- ○ Stroke
- ○ Thyroid disease
- ○ Ulcerative colitis
- ○ Other conditions not listed above (Please specify)

1. ____________________

2. ____________________

SECTION C: PATIENT ASTHMA CONTROL ASSESSMENT

(Using the validated Asthma Control Test (ACT) - 5 item) ©

Copyright 2002 by QualityMetric Incorporated.ASTHMA CONTROL TEST is a trademark of QualityMetric Incorporated

C1. In the past 4 weeks, how much of the time did your asthma keep you from getting as much done at work, school or at home?

○ All of the time ○ Most of the time ○ Some of the time ○ A little of the time ○ None of the time

C2. During the past 4 weeks, how often have you had shortness of breath?

○ More than once per day ○ Once per day ○ 3 to 6 times per week ○ Once or twice a week ○ Not at all

C3. During the past 4 weeks, how often did your asthma symptoms (wheezing, coughing, shortness of breath, chest tightness or pain) wake you up at night or earlier than usual in the morning?

○ 4 or more nights a week ○ 2 or 3 nights a week ○ Once a week ○ Once or twice ○ Not at all

C4. During the past 4 weeks, how often have you used your reliever inhaler or nebulizer medication (such as salbutamol)?

○ 3 or more times per day ○ 1 or 2 times per day ○ 2 or 3 times per week ○ Once a week or less ○ Not at all

C5. How would you rate your asthma control during the past 4 weeks?

○ Not controlled at all ○ Poorly controlled ○ Somewhat controlled ○ Well controlled ○ Completely controlled

SECTION D: PATIENT ASTHMA QUALITY OF LIFE ASSESSMENT

(Using the validated Sydney Asthma Quality of Life Questionnaire (AQLQ-S) - 20 item) ©

D1.

**In the past 4 weeks, how much have the following affected you?** (Please indicate the response to each statement, which closely applies to you over the past four weeks)	Not At All	Mildly	Moderately	Severely	Very Severely
I have been troubled by episodes of shortness of breath.	○	○	○	○	○
I have been troubled by wheezing attacks.	○	○	○	○	○
I have been troubled by tightness in the chest.	○	○	○	○	○
I have been restricted in walking down the street on level ground or doing light housework because of asthma or shortness of breath.	○	○	○	○	○
I have been restricted in walking up hills or doing heavy housework because of asthma or shortness of breath.	○	○	○	○	○
I have felt tired or a general lack of energy.	○	○	○	○	○
I have been unable to sleep at night.	○	○	○	○	○
I have felt sad or depressed.	○	○	○	○	○
I have felt frustrated with myself.	○	○	○	○	○
I have felt anxious, under tension or stressed.	○	○	○	○	○
I have felt that asthma or shortness of breath is preventing me from achieving what I want from life.	○	○	○	○	○
Asthma or shortness of breath has interfered with my social life.	○	○	○	○	○
I have been limited in going to certain places because they are bad for my asthma.	○	○	○	○	○
I have been limited in going to certain places because I have been afraid of getting an asthma attack and not being able to get help.	○	○	○	○	○
I have been restricted in the sports, hobbies or other recreations I can engage in because of my asthma or shortness of breath.	○	○	○	○	○
I have felt generally restricted.	○	○	○	○	○
I have felt that asthma is controlling my life.	○	○	○	○	○
I have been worried about my present or future health because of asthma.	○	○	○	○	○
I have been worried about asthma shortening my life.	○	○	○	○	○
I have felt dependent on my asthma inhalers.	○	○	○	○	○

SECTION E: PATIENT MEDICATION ADHERENCE ASSESSMENT

(Using the validated Adherence Starts with Knowledge (ASK-12) Adherence Barrier Survey - 12 item) ©

E1. Think about the medicines you take. Mark **ONE** answer for each item.

INCONVENIENCE/FORGETFULNESS<br>*LIFESTYLES*	Strongly Agree	Agree	Neutral	Disagree	Strongly Disagree
I just forget to take my medicines some of the time.	○	○	○	○	○
I run out of my medicine because I don't get repeats on time.	○	○	○	○	○
Taking medicines more than once a day is inconvenient.	○	○	○	○	○

E2. Think about the medicines you take. Mark **ONE** answer for each item.

TREATMENT BELIEFS<br>*ATTITUDES and BELIEFS*	Strongly Agree	Agree	Neutral	Disagree	Strongly Disagree
I feel confident that each one of my medicines will help me.	○	○	○	○	○
I know if I am reaching my health goals.	○	○	○	○	○
HELP FROM OTHERS					
I have someone I can call with questions about my medicines.	○	○	○	○	○
TALKING WITH HEALTHCARE TEAM					
My doctor/nurse and I work together to make decisions.	○	○	○	○	○

E3. Have you.....

BEHAVIOUR<br>*TAKING MEDICINES*	In the last Week	In the last Month	In the last 3 Months	More Than 3 Months Ago	Never
Taken a medicine more or less often than prescribed?	○	○	○	○	○
Skipped or stopped taking a medicine because you didn't think it was working?	○	○	○	○	○
Skipped or stopped taking a medicine because it made you feel bad?	○	○	○	○	○
Skipped, stopped, not refilled, or taken less medicine because of the cost?	○	○	○	○	○
Not had your medicine with you when it was time to take it?	○	○	○	○	○

This material was developed by GlaxoSmithKline - © 2008 The GlaxoSmithKline Group of Companies.
All rights reserved - Used with permission

SECTION F: ASTHMA KNOWLEDGE ASSESSMENT

(Using the validated Consumer Asthma Knowledge Questionnaire (CQ) - modified 10 item)

© Vicky Kritikos et al, 2005

The following series of statements relates to your knowledge and opinions about asthma.

F1. You can become addicted to asthma medications if you use them all the time.

- ○ True
- ○ False

F2. An asthma action plan can prevent hospitalisations due to asthma.

- ○ True
- ○ False

F3. When you know that you are going to be exposed to something that triggers your asthma, you should take the recommended medication just before exposure.

- ○ True
- ○ False

F4. When you know that you are going to be exposed to something that triggers your asthma, you should wait until you develop symptoms before taking medication.

- ○ True
- ○ False

F5. Side effects are less likely with inhaled medications than with tablets.

- ○ True
- ○ False

F6. With preventer medications, it does not matter if some doses are missed or if you go on and off them.

- ○ True
- ○ False

F7. If you get a cold or flu, you should increase your asthma medications.

- ○ True
- ○ False

F8. Some medications can trigger asthma attacks.

- ○ True
- ○ False

F9. Going from a cold to a hot environment can trigger asthma, but going from a hot to a cold environment does not trigger asthma.

- ○ True
- ○ False

F10. Parents should give "reliever medication" to a child as soon as they recognise the first sign of asthma.

- ○ True
- ○ False

SECTION G: PATIENT BELIEFS ABOUT ASTHMA CONTROL

(Using the Perceived Control of Asthma Questionnaire (PCAQ) 11 item)
© Patricia Katz, 2002

G1.

**PERCEPTION/BELIEF** (Please indicate your level of agreement with the following statements)	Strongly Agree	Agree	Neutral	Disagree	Strongly Disagree
I can reduce asthma by staying calm and relaxed.	○	○	○	○	○
Too often, my asthma just seems to hit me out of the blue.	○	○	○	○	○
If I do all the right things, I can successfully manage my asthma.	○	○	○	○	○
I can do a lot of things myself to cope with my asthma.	○	○	○	○	○
When I manage my personal life well, my asthma does not affect me as much.	○	○	○	○	○
I have considerable ability to control my asthma.	○	○	○	○	○
I would feel helpless if I couldn't rely on other people for help when I'm not feeling well from asthma.	○	○	○	○	○
No matter what I do, or how hard I try, I just can't seem to get relief from my asthma.	○	○	○	○	○
I am coping effectively with my asthma.	○	○	○	○	○
It seems as though fate and other factors beyond my control affect my asthma.	○	○	○	○	○
Asthma is controlling my life.	○	○	○	○	○

Thank you for your time.

Approval for the conduct of this study has been granted by the Human Research Ethics Committee, University of Western Australia, and will be carried out in a manner conforming to the principles set out by the National Health and Medical Research Council (NHMRC).

All data collected will be kept confidential and subsequently destroyed. All reports or articles will be de-identified and your identity will be safeguarded at all times. Only those directly involved in the study will have access to your personal details.

If you would like a report of the study on completion please ask the investigator and the report that may consist of positive and negative findings will be forwarded to you.

Any person with concerns or complaints about the conduct of a research study can contact:
Human Research Ethics Office at The University of Western Australia on (08) 6488 3703
or by emailing to hreo-research@uwa.edu.au
